# Anaplastic Lymphoma Kinase (ALK) in Posterior Cranial Fossa Tumors: A Scoping Review of Diagnostic, Prognostic, and Therapeutic Perspectives

**DOI:** 10.3390/cancers16030650

**Published:** 2024-02-02

**Authors:** Danai-Priskila V. Mousa, Georgios Mavrovounis, Dionysios Argyropoulos, George Stranjalis, Theodosis Kalamatianos

**Affiliations:** 1 Department of General Surgery, Penteli Children’s Hospital, 15236 Athens, Greece; danaemusa@gmail.com; 2 Department of Neurosurgery, Faculty of Medicine, School of Health Sciences, University of Thessaly, 41334 Larissa, Greece; gmavrovounis@gmail.com; 3 Department of Neurosurgery, Evangelismos Hospital, School of Medicine, Faculty of Health Sciences, National and Kapodistrian University of Athens, 10676 Athens, Greece; stranjal@otenet.gr; 4 Department of Psychiatry, Eginition Hospital, National and Kapodistrian University of Athens, 11528 Athens, Greece; dionargi66@gmail.com

**Keywords:** Anaplastic Lymphoma Kinase (ALK), medulloblastoma, histiocytosis, central nervous system, ALK inhibition

## Abstract

**Simple Summary:**

Anaplastic Lymphoma Kinase (ALK) is a protein linked to cancer growth. A review of scientific studies was conducted to understand ALK’s role in certain brain tumors, particularly those not originating from glial cells (supportive cells in the brain) and located in the lower back part of the brain. From an initial pool of 992 studies, 16 were found to be relevant. These studies focused on 55 cases of tumors displaying ALK presence or ALK alterations, including medulloblastoma, lymphoma, histiocytosis, and other rare tumors. Studies mainly used tissue analysis and genetic testing to study ALK. Findings suggest that examining ALK can help in diagnosing and predicting the outcome of some of these brain tumors, especially medulloblastoma. Interestingly, many cases of brain histiocytosis (a rare condition) with ALK changes were found in this area. These findings point to the potential benefits of targeting ALK in treating certain brain tumors, a promising area for future research.

**Abstract:**

Anaplastic Lymphoma Kinase (ALK) has been implicated in several human cancers. This review aims at mapping the available literature on the involvement of ALK in non-glial tumors localized in the posterior cranial fossa and at identifying diagnostic, prognostic, and therapeutic considerations. Following the PRISMA-ScR guidelines, studies were included if they investigated ALK’s role in primary CNS, non-glial tumors located in the posterior cranial fossa. A total of 210 manuscripts were selected for full-text review and 16 finally met the inclusion criteria. The review included 55 cases of primary, intracranial neoplasms with ALK genetic alterations and/or protein expression, located in the posterior fossa, comprising of medulloblastoma, anaplastic large-cell lymphoma, histiocytosis, inflammatory myofibroblastic tumors, and intracranial myxoid mesenchymal tumors. ALK pathology was investigated via immunohistochemistry or genetic analysis. Several studies provided evidence for potential diagnostic and prognostic value for ALK assessment as well as therapeutic efficacy in its targeting. The available findings on ALK in posterior fossa tumors are limited. Nevertheless, previous findings suggest that ALK assessment is of diagnostic and prognostic value in medulloblastoma (WNT-activated). Interestingly, a substantial proportion of ALK-positive/altered CNS histiocytoses thus far identified have been localized in the posterior fossa. The therapeutic potential of ALK inhibition in histiocytosis warrants further investigation.

## 1. Introduction

Anaplastic Lymphoma Kinase (ALK), a receptor tyrosine kinase, is part of the insulin receptor superfamily and shares notable similarities with leukocyte tyrosine kinase (LTK) [1]. The ALK gene in humans is located on the chromosomal segment 2p23. It encodes a polypeptide chain that is composed of 1620 amino acids. Following several post-translational modifications, this polypeptide becomes the mature ALK protein, with an approximate molecular weight ranging from 200 to 220 kDa [2,3]. The mature form of ALK has the typical structure seen in receptor tyrosine kinases. It consists of three distinct domains: an extracellular domain that binds ligands, comprising 1030 amino acids; a transmembrane domain of 28 amino acids; and an intracellular tyrosine kinase domain, which includes 561 amino acids [4]. A specific motif (Tyr1278, Tyr1282, and Tyr1283), crucial for auto-phosphorylation and enzymatic activity, is situated within the activation loop of the intracellular domain [5,6]. ALK is activated solely when a ligand triggers homodimerization, while deactivation is achieved by dephosphorylation by the protein tyrosine phosphatase receptor beta and zeta 1 complex (PTPRB/PTPRZ1) [7]. Even though substances such as midkine and pleiotrophin are known to activate ALK in mammals, it is important to note that these ligands are not exclusively specific to ALK [8,9,10]. Recently, Augmentor-α (AUG-α, also known as FAM150B) and Augmentor-β (AUG-β, FAM150A) have been identified as specific ligands for ALK [11,12]. AUG-α binds to ALK with high affinity, activating it with substantial potency. This ligand acts as a dual-specific activator for both ALK and LTK, whereas AUG-β shows more specificity towards LTK, only weakly interacting with ALK [11,12].

ALK was initially characterized as a novel tyrosine phosphoprotein in cell lines of anaplastic large-cell lymphoma (ALCL) in 1994 [13,14]. Its identification revealed a chimeric protein formed through a translocation event between chromosomes (2;5)(p23:q35), giving rise to a previously unexplored fusion protein named nucleophosmin (NPM)–ALK. This fusion comprises the N-terminal segment of the NPM protein and the kinase domain of a then unknown tyrosine kinase protein, subsequently named ALK after the associated disease [15]. The activated ALK triggers various signaling pathways that contribute to cell growth, prevention of cell death, and immune evasion [16]. Key pathways activated by ALK are Janus kinase/signal transducers and activators of transcription (JAK/STAT), rat sarcoma/mitogen activated protein kinase (RAS/MAPK), phosphatidylinositol 3-kinase/Ak strain transforming (PI3K/AKT), and Phosphoinositide phospholipase C (PLC-γ) [17]. Approximately 10% of cases exhibit different ALK-partner gene variants, such as tropomyosin 3 (TPM3), 5-aminoimidazole-4-carboxamide ribonucleotide formyltransferase/IMP cyclohydrolase (ATIC), Clathrin heavy chain (CTLC), myosin heavy chain 9 (MYH9). Unlike variant fusion proteins, NPM1–ALK is uniquely expressed in both the nucleus and the cytoplasm [18].

**Figure 1:**
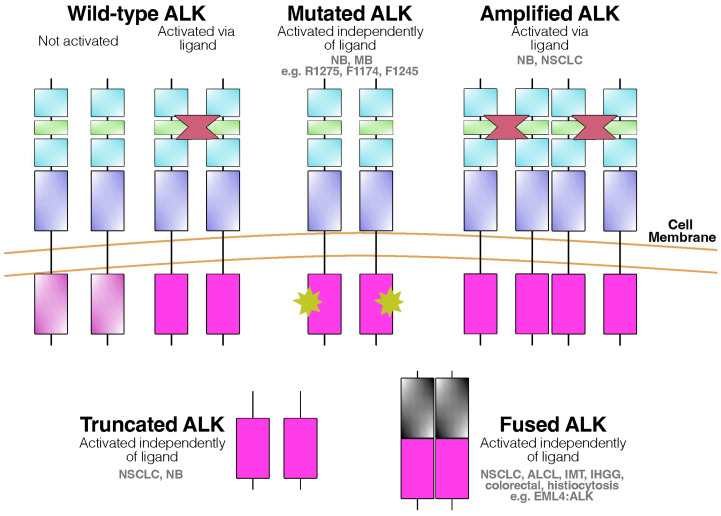
ALK alterations in cancer. Adapted from [27]. Abbreviations: ALK—Anaplastic Lymphoma Kinase; NB—neuroblastoma; MB—medulloblastoma; NSCLC—non-small-cell lung cancer; ALCL—anaplastic large-cell lymphoma; IMT—inflammatory myelofibroblastic tumor; IHGG—infantile hemispheric glioma.

Since the initial discovery of the NPM–ALK fusion protein in ALCL patients [13,14], ALK fusion proteins have been identified in various other tumors, such as inflammatory myofibroblastic tumors (IMTs) [19] and non-small-cell lung cancer (NSCLC) [20,21]. Notably in NSCLC, five recurrent chromosomal translocations involving the ALK gene have been reported, resulting in fusion proteins such as echinoderm microtubule-associated protein-like 4 (EML4)–ALK (120 kDa, inv(2)(p21;p23); 13 variants), tyrosine receptor kinase-fused gene (TFG)–ALK (113 kDa, t(2;3)(p23;q21)), kinesin family 5B (KIF5B)–ALK (t(2;10)(p23;p11)), kinesin light chain 1 (KLC1)–ALK (t(2;14)(p23;q32)), and protein tyrosine phosphatase non-receptor type 3 (PTPN3)–ALK (t(2;9)(p23;q31)) [5]. The most prevalent EML4–ALK fusion occurs with a frequency ranging from 0.1% to 7.9%, encompassing 13 translocation variants [15]. Additionally, full-length ALK expression has been reported in cell lines and tumors, indicating oncogenic progression through overexpression [2,22,23] or gain-of-function mutations, as recently observed in neuroblastoma cases [24]. Finally, a truncated form of ALK, lacking the extracellular domain, has also been detected in neuroblastoma cases [25,26]. Figure 1 illustrates ALK alterations in cancer cells.

The complete ALK is found in a variety of tumor types. For example, the full-length ALK receptor protein is present in cell lines and specimens from tumors such as neuroblastomas, neuroectodermal tumors [28,29], and glioblastomas [23,28]. The first successful cloning of full-length ALK cDNA utilized a cDNA library from an Rh30 rhabdomyosarcoma cell line [28] and further studies have confirmed its presence in certain rhabdomyosarcoma tumors [28,30]. Furthermore, anti-ALK immunoreactivity has been observed in other types of cancer, including breast carcinoma, malignant peripheral nerve-sheath tumors, and lipogenic tumors [31]. However, in these tumors, it remains unclear whether the immunoreactivity results from the full-length or fusion forms of ALK [32]. While strong evidence links full-length ALK expression to neuroblastoma, the significance of ALK receptor expression in other tumor types remains poorly understood. Importantly, ALK protein expression in neuroblastomas correlates with a poorer prognosis [33,34]. In contrast, published data related to glial tumors suggest that the role of activating ALK fusions or mutations in the development of most primary central nervous system (CNS) tumors is minimal, with the exception of the extremely rare cases of infant-type hemispheric gliomas [35]. Based on the aforementioned evidence, routine testing for ALK changes is generally not advised. Consequently, the effectiveness of treatments targeting ALK is considered limited for gliomas, glioneuronal and neuronal tumors in adults, and in the majority of CNS tumors found in children (ESCAT IIIA). According to the latest guidelines from the European Association of Neuro-Oncology (EANO), the consideration of ALK-specific treatments is recommended only within clinical trials or prospective registries, and only after all standard treatment options have been tried [36].

Anaplastic Lymphoma Kinase (ALK) inhibitors have revolutionized the treatment of ALK-positive cancers, particularly non-small cell lung cancer (NSCLC) [37]. Starting with Crizotinib, the first-generation inhibitor, subsequent generations like Ceritinib, Alectinib, Brigatinib, Ensartinib, and Lorlatinib have progressively improved efficacy and overcome resistance issues. Lorlatinib, a third-generation inhibitor, is notable for its CNS penetration and effectiveness in patients with prior resistance to earlier inhibitors [37]. Recent developments in ALK inhibitors have introduced novel drugs like Iruplinalkib, XMU-MP-5, APG-2449, and Repotrectinib, targeting various aspects of the ALK pathway and showing promising results in both preclinical and early clinical trials. Additionally, emerging fourth-generation inhibitors such as TPX-0131 and NVL-655 are being evaluated for their efficacy against ALK-positive advanced/metastatic NSCLC and other solid tumors [37,38,39].

The posterior cranial fossa, an anatomically complex region located at the skull’s base, contains brain structures such as the brainstem, which is vital for the control of essential bodily functions (e.g., breathing, heart rate, sleep) and the cerebellum which plays a pivotal role in the regulation/coordination of movement [40]. In children with congenital neuroblastoma, certain germline ALK mutations have been linked to abnormal morphology of posterior cranial fossa structures, including the brainstem [41,42]. These findings have been previously taken as evidence for a potential role of ALK in the normal development of posterior fossa structures. Some authors have also suggested that exploring the implications of endogenous expression of mutant ALK on both neurological function and the anatomic development of the pons, medulla, and motor neurons may be of considerable interest [30].

Surgery of the posterior fossa can carry a higher risk of complications compared to supratentorial surgery [43]. The increased risk is often attributed to the complex nature of the posterior fossa compartment and the technically challenging surgical approaches to the region. These often result in a variety of complications, such as cerebrospinal fluid leaks, cranial nerve palsy, and hemorrhage, affecting overall patient outcomes [43,44]. In neurosurgical oncology operations of the posterior fossa, postoperative residual disease is a common phenomenon, with rates as high as 70% [45]. An exceptionally devastating complication most commonly linked to posterior fossa pediatric oncology operations (up to 25% of cases) is cerebellar mutism [46]. The condition is characterized by speech loss or reduction and is commonly accompanied by emotional alterations and motor coordination difficulties, often resulting in life-long disability [46,47]. Consequently, treatment plans usually combine surgery with chemoradiation treatment to improve patient outcomes [48]. Identifying novel pharmacological targets for these tumors could allow for improved, targeted approaches to the disease and enhance patient outcomes.

In this scoping review, we will provide an overview of the evidence indicating diagnostic/prognostic value of ALK expression and of ALK genetic/molecular alterations in primary CNS, non-glial tumors presenting in the posterior cranial fossa. We will also map the evidence for the therapeutic potential of ALK targeting in these tumors.

## 2. Materials and Methods

The Preferred Reporting Items for Systematic Reviews and Meta-Analyses Extension for Scoping Reviews (PRISMA-ScR) was followed for this scoping review (Supplementary File S1) [49]. A protocol for the review steps was published online on Open Science Framework Registries prior to review initiation. The full protocol is available at https://osf.io/sdm2b (accessed on 28 January 2024).

### 2.1. Literature Search

The PubMed and Scopus databases were searched by two independent researchers (D.-P.V.M., D.A.). Any conflicts between the researchers were resolved through consultation with a third reviewer (G.M.). The algorithms used included the terms “Anaplastic Lymphoma Kinase”, “ALK”, “intracranial”, “intra-cranial”, “central nervous”, “CNS”, “Central Nervous System”, “Central Nervous System Neoplasms”, “brain”, “cranial”, “cerebr*”, “encephal*”, “spinal cord”, “spinal cord neoplasms”, and specific non-glial tumor names, combined with the Boolean operators “AND” and “OR”. Non-glial tumors were selected according to the World Health Organization Classification of Tumors of the Central Nervous System (5th Edition) [50].

Independent searches were performed for each non-glial tumor type utilizing the aforementioned terms. Supplementary File S2 presents the algorithms used for database search. The last literature search was performed on 30 November 2023. Furthermore, the bibliographies of all included articles were manually reviewed to identify additional studies.

After removal of duplicates, titles and abstracts were screened for relevance. Subsequently, the full texts of potentially relevant articles were reviewed to identify studies that fulfilled the specific inclusion criteria. Any conflicts regarding study selection were resolved through discussion.

### 2.2. Study Selection

Inclusion criteria were: (1) randomized controlled trials, observational studies, and case reports; (2) investigating the role of ALK (3) in non-glial tumors (as per the 2021 WHO Classification of Intracranial Tumors); (4) presenting with primary CNS lesions (5) in the posterior cranial fossa of patients. Only studies in English were considered eligible for inclusion.

We excluded reviews, systematic reviews, and meta-analyses, letters to the editor, and short communications. Animal studies and gray literature were not included.

### 2.3. Data Extraction

Two independent researchers (D.-P.V.M., D.A.) extracted the following information in a pre-designed Excel spreadsheet: name of first author, year of publication, study type, number of patients, age, sex, symptoms leading to diagnosis, type of tumor, exact location of tumor, tumor size, history of other malignancies, therapeutic approach relating to ALK, other therapeutic approaches not related to ALK, ALK analysis method, ALK type of pathology, and main conclusions of the study. 

## 3. Results

**Figure 2:**
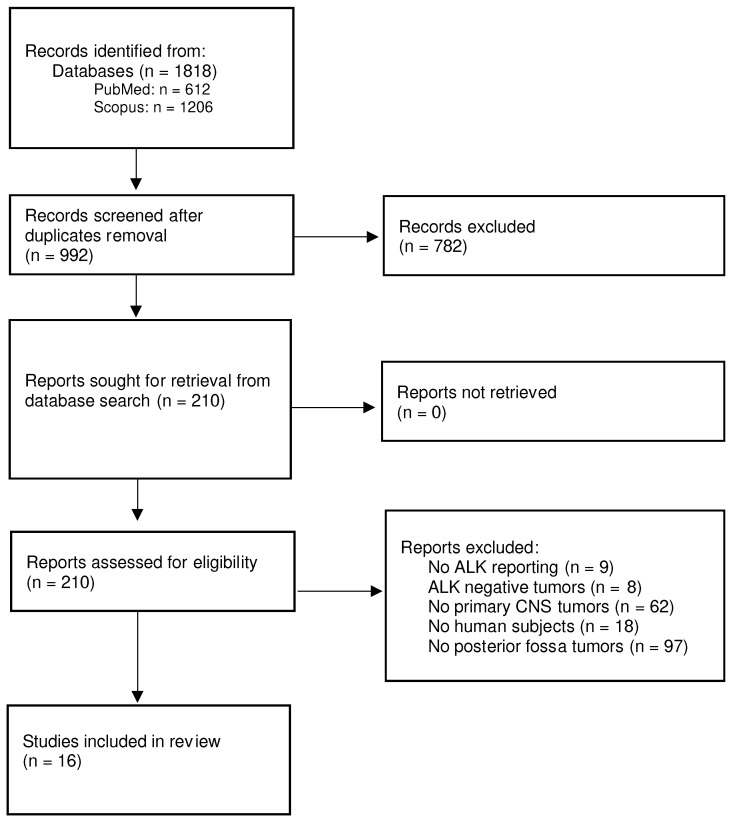
Flowchart for study selection.

Our search identified 992 relevant citations which were subsequently screened, resulting in 210 articles selected for full-text review. After full text review, we identified 16 pertinent studies in which data extraction was possible, published between 1995 and 2023 (Figure 2). The 16 articles included a total of 55 cases, in which the primary, intracranial ALK-positive or ALK-altered tumor was located in the posterior fossa (brainstem, pons, cerebellum, or cerebellopontine angle-CPA). The basic characteristics of the studies are presented in Table 1.

ALK pathology was assessed using either immunohistochemistry (IHC), fluorescent in situ hybridization (FISH), or via additional genetic analysis (sequencing) for the presence ALK gene alterations. All identified articles were case reports and small case series.

Out of the 16 articles, 5 were related to medulloblastoma, 6 to ALCL, and the remaining 5 articles covered IMT (n = 1), histiocytosis (n = 2), and IMMT (n = 2).

Regarding the cases associated with medulloblastoma, a total of 40 cases were identified in the posterior cranial fossa, among which the tumor was located in the CPA, the cerebellar hemispheres, the posterior fossa midline, or the fourth ventricle. The male/female ratio was 16:19 (gender was not reported in five cases), and the patients were primarily children aged <1–16 years. Another study by Li et al. provided diagnostic accuracy data regarding ALK RNA expression in medulloblastoma [51]. However, it was excluded from our review as it did not provide raw data regarding their cases. 

A total of five single-system histiocytosis cases were observed in the posterior cranial fossa. Of these, three were located in the cerebellum and two were located in the medulla. The male/female ratio was 1:4, with the age at diagnosis ranging from 9 months to 7 years old. For ALCL, a total of six cases were observed in the posterior cranial fossa. The lesions were located in the cerebellum, the brainstem, and the associated dura. The male/female ratio was 2:3 (gender was not reported in one case), and the ages of the patients ranged from 4.5 to 43 years (Table 1). Finally, four additional cases were identified, a 65-year-old woman with IMMT in the right cerebellar hemisphere, a 10-year-old girl with IMMT in the left cerebellar hemisphere, and one IMT case in the cerebellar region of a 30-year-old male (Table 1). 

**Table 1:** Basic characteristics of included studies.

Author YOP Tumor of Interest	Study Design Number of Patients Included in Review	Age (Years) Gender (M: Male, F: Female)	Exact Location of Posterior Fossa Tumor	Therapeutic Approach Related to ALK Other Therapeutic Approaches Not Related to ALK	ALK Analysis Method with Molecular Targets	ALK Type of Pathology
Ahrendsen et al. 2022 [52] ALCL	Case Series 1	37 M	Infratentorial Compartments (including cerebellum)	- Surgery, Intrathecal Methotrexate	IHC, sequencing (447 gene exons, 191 introns across 60 genes)	ALK-positive, no genetic alterations detected
Geetha et al. 2014 [53] ALCL	Case Report 1	19 M	Right Cerebellar Hemisphere	- Surgery, Chemotherapy	IHC	ALK-positive
Havlioglu et al. 1995 [54] ALCL	Case Report 1	4.5 F	Multifocal brain, Brainstem, Spinal Cord	- Chemotherapy, Radiation	IHC	ALK-positive
Menon et al. 2015 [55] ALCL	Case Series 1	43 Μ	Multiple meningeal lesions (Right Cerebellum, Medulla involvement)	- NA	IHC	ALK-positive
Rudresha et al. 2017 [56] ALCL	Case Series 1	NA NA	Cerebellum	- High-dose methotrexate, Radiation	IHC	ALK-positive
Strosberg et al. 2021 [57] ALCL	Case Report 1	29 F	Brainstem	- Chemotherapy, Methotrexate	IHC	ALK-positive
Hojo et al. 2023 [58] IMMT	Case Report 1	10 F	Left Cerebellar Hemisphere	- Surgery	IHC	ALK-positive
Kambe et al. 2021 [59] IMMT	Case Report 1	65 F	Right Cerebellar Hemisphere	- Surgery	IHC	ALK-positive
Kemps et al. 2022 [60] Histiocytosis	Case Series 4	0.75, 2.5, 3, 7 F (n = 3) M (n = 1)	Cerebellum (n = 2) Medulla (n = 2)	Alectinib (n = 1) Chemotherapy (n = 2) Corticosteroids, Surgery (n = 1) NA (n = 1)	IHC, FISH, sequencing	ALK-positive IHC (n = 4) ALK-FISH positive (n = 1) KIF5B–ALK fusion (n = 3)
Lucas et al. 2019 [61] Histiocytosis	Case Report 1	7 F	Cerebellar Vermis	- Surgery	IHC, sequencing (Sequencing: RNA assay targeting 53 genes)	KIF5B–ALK fusion
Swain et al. 2008 [62] IMT	Case Report 1	30 M	Cerebellum	- Radiation, Surgery	IHC, FISH (FISH: ALK rearrangement in 4% of tumor cells)	ALK negative IHC ALK-FISH positive
Coco et al. 2012 [63] MB	Case Series 4	Pediatric NA	Cerebellum	- -	PCR, sequencing a. Sequencing for ALK exons 20–28 b. Quantitative PCR for mRNA expression	ALK mutation 3605delG in exon 23 of ALK gene (n = 1) ALK mRNA overexpression (n = 3)
Łastowska et al. 2017 [35] MB WNT (n = 19) SHH (n = 2) Type 3 (n = 1) Type 4 (n = 1) Not classified (n = 2)	Case Series 25	<1–14 F (n = 13) M (n = 12)	Cerebellopontine Angle (n = 3) Cerebellar Hemisphere (n = 1) Cerebellar Midline (n = 21)	- -	IHC, sequencing	All cases: ALK-positive Additional methods: NanoString, CTNNB1 mutation (n = 9) NanoString (n = 9) CTNNB1 mutation, monosomy 6 (n = 1) Nuclear b-catenin, monosomy 6 (n = 2) MAGIC MB128 (n = 2) GAB1+, YAP1+ (n = 1)
Łastowska et al. 2019 [64] MB	Case Series 7	3–16 F (n = 4) M (n = 2) NA (n = 1)	Cerebellopontine Angle (n = 2) Cerebellar Midline (n = 4) NA (n = 1)	- -	IHC, DNA (next generation, Sanger) sequencing	ALK-positive (n = 7) APC variant detected (n = 1)
Trubicka et al. 2016 [65] MB	Case Report 1	10 M	Posterior Fossa Midline	- Surgery	IHC	ALK-positive
Yan et al. 2016 [34] MB WNT (n = 1) SHH (n = 1) Mixed − WNT/SHH (n = 1)	Case Series 3	1.8, 5.5, 12.8 F (n = 2) M (n = 1)	Posterior Fossa - 4th Ventricle (n = 2) Right Cerebellar Hemisphere (n = 1)	- Surgery, Radiation, Chemotherapy (n = 2) Surgery alone (n = 1)	IHC, DNA sequencing, FISH (DNA sequencing: ALK exons 23, 25 FISH: >15% of cells having split signals)	ALK-positive ALK-FISH negative

Abbreviations: YOP—year of publication; ALK—Anaplastic Lymphoma Kinase; ALCL—anaplastic large-cell lymphoma; IHC—immunohistochemistry; NA—not available; IMMT—intracranial myxoid mesenchymal tumor; FISH—fluorescence in situ hybridization; MB—medulloblastoma; PCR—polymerase chain reaction; WNT—wingless-related integration site; SHH—sonic hedgehog; GAB1—GRB2-associated-binding protein 1; YAP1—yes-associated protein 1; APC—adenomatous polyposis coli.

## 4. Discussion

### 4.1. Medulloblastoma

Medulloblastoma, a prototypical posterior fossa tumor, is the most common malignant tumor in the pediatric population, with at least four distinct molecular subgroups identified, namely wingless (WNT), sonic hedgehog (SHH), Group 3, and Group 4 [50]. Based upon the recent 2021 WHO Classification of Tumors of the Central Nervous System, the presence of each subgroup (including different subtypes within the same subgroup) can have different clinical, molecular, therapeutic, and prognostic implications [50]. WNT-activated medulloblastomas are typically localized in the cerebellar midline or CPA region and have better prognosis when compared to the other types [64]. In contrast, SHH medulloblastomas are typically located in the cerebellar hemispheres and have worse prognosis. Non-WNT/non-SHH medulloblastomas are characterized by their cerebellar midline location [64]. The difference in prognosis has led to efforts to decrease the intensity of therapy in low-risk medulloblastomas, in order to minimize the short- and long-term side effects of therapeutic interventions [66,67]. However, identifying WNT-activated medulloblastomas can be challenging even when following specific guidelines [50,64]. Consequently, there are ongoing efforts to identify further specific markers of good prognosis in medulloblastomas.

Previous studies investigating hotspot mutations in exons 23 and 25 estimate that ALK alterations occur in around 1–2% of medulloblastoma tumors [34]. Additional findings suggest that ALK protein expression alone could be an indicator of a favorable prognosis for medulloblastoma patients and that ALK expression could be a crucial marker for identifying WNT-activated medulloblastoma tumors, including those in the cerebellopontine angle (CPA) [35,64]. 

Thus, survival analysis indicates that the presence of ALK protein expression alone, when detected immunohistochemically in over 50% of tumor cells, has prognostic value even in the absence of established molecular profiling of the tumors [35]. Interestingly, long-term survival was reported for patients with ALK+/unclassified tumors or ALK+/Group 4 tumors [35]. Immunohistochemical detection of ALK has been postulated to be of significant value in the identification of WNT-activated medulloblastomas, complementing methods like CTNNB1 gene mutation and β-catenin nuclear reaction analysis [64]. In line with the aforementioned evidence, Li et al. reported that ALK RNA expression, alongside FGFR1 RNA expression, exhibited excellent diagnostic accuracy for the identification of WNT-activated medulloblastomas [51]. Notably, in one of the studies, among the cohort of ALK-positive patients, one individual who exhibited a low-level ALK copy number gain experienced disease recurrence merely three months after undergoing a gross total resection [63]. 

Overall, ALK’s role in medulloblastoma may differ from its function in other tumors in children. Although the presence of ALK is associated with a worse prognosis in neuroblastoma and rhabdomyosarcoma, the same does not apply to medulloblastomas. Given the significant expression of the intracellular fragment of the protein in these tumors, targeting ALK with inhibitors remains a feasible option, especially in the uncommon instances of relapse in WNT tumors [29]. 

In pediatric patients, distinguishing medulloblastomas from other primary malignant brain tumors that originate in the posterior fossa is a complex task. This group of challenging malignancies includes atypical teratoid rhabdoid tumors, choroid plexus carcinomas, and anaplastic ependymomas. These tumors, characterized by their high cellularity, may exhibit small, round, poorly differentiated cells that bear a resemblance to those found in medulloblastomas [64]. Lastowska et al. suggest that ALK protein expression may be a distinctive diagnostic marker for differentiating WNT-activated medulloblastomas from other high-grade pediatric tumors in the posterior fossa that share similar histological features [64].

### 4.2. Histiocytosis

Histiocytosis is an umbrella term for diseases characterized by the proliferation of cells originating from macrophages, dendritic cells, or monocytes [68]. This category includes over 100 subtypes, with each presenting distinct clinical and pathological features [68]. Initially thought to be autoimmune, inflammatory disorders, they are now considered hematologic malignancies of clonal origin [69]. Histiocytoses can affect any organ in the body and often present as multisystemic disorders [69]. The CNS is one of the most commonly involved organs in multisystemic disease [70], but it can also be affected in single-organ disease [71]. CNS histiocytosis can present both in the supratentorial and the infratentorial (posterior fossa) region [72]. 

ALK alterations in children and adults with single-organ or multisystemic histiocytosis of the CNS have been consistently reported in the last quinquennium [60]. A recent study, which represents the largest series of ALK-altered histiocytosis cases to date, highlighted the frequent involvement of the nervous system in these conditions [60]. Interestingly, 5 of the 11 single-system ALK-positive or ALK-altered neurologic cases available in the literature were located in the posterior fossa [60,61,73], highlighting a topographical predilection for posterior fossa structures such as the cerebellum and the brainstem. 

The most commonly reported genetic mutation in the literature is the KIF5B–ALK fusion, although other fusions, such as CLTC–ALK, TPM3–ALK, TFG–ALK, EML4–ALK, and DCTN1–ALK, present less frequently [60,61]. Indeed, 9 of the 11 identified single-system neurologic cases were reported to exhibit the KIF5B–ALK fusion. Overall, only three of them received therapy with ALK inhibition (alectinib, lorlatinib), probably due to the excellent response they had to surgical intervention. More specifically, four of the five posterior fossa tumors identified in our review presented the KIF5B–ALK fusion. Notably, only one of them received therapy with ALK inhibition [60]. The rest of the patients were treated with surgical resection and/or corticosteroids and other chemotherapy regimens [60,61]. Kemps et al. reported that patients with neurological involvement receiving ALK inhibition therapy experienced a ‘dramatic and durable’ response rate of 100%, notably higher than the 50% response rate observed in patients treated with other chemotherapy regimens [60]. 

Based on the findings in the literature, histiocytosis cases, especially those with neurological involvement, should be examined for ALK positivity or ALK alterations. Kemps et al. propose that ALK-positive histiocytosis cases should undergo further molecular testing to confirm ALK rearrangement, as immunoreactivity is not always enough to predict response to treatment [60]. Currently, it is unclear whether ALK-inhibition chemotherapy should be used as a first- or second-line treatment; the optimal treatment regimen is another issue that remains to be addressed. 

### 4.3. Anaplastic Large-Cell Lymphomas (ALCL) 

Primary CNS lymphomas are rare, accounting for around 4% of all intracranial neoplasms [74]. They are mainly diffuse large B-cell lymphomas confined to the central nervous system, including the brain and spinal cord [75]. Primary CNS lymphomas typically affect adults in their sixth decade of life, but can also occur in immunocompromised individuals. Symptoms vary based on the tumor location and can include headaches and neurological deficits. Treatment primarily involves high-dose methotrexate-based chemotherapy, sometimes in combination with rituximab [75].

Non-CNS lymphomas with ALK expression generally have better prognosis in comparison to their ALK-negative counterparts, as they tend to respond well to chemotherapy and to remain in remission [76,77]. Primary CNS ALCL, characterized by ALK expression, predominantly affects children and young adults and occurs more frequently in male patients. Most tumors are solitary (73.5% of cases), with 26.5% of patients presenting multifocal disease within the CNS [52]. The majority of tumors are located in the supratentorial compartment, with fewer cases in the infratentorial (including the posterior fossa) or both compartments. Dural/leptomeningeal involvement alone or in combination with parenchymal lesions is frequently observed [52].

Our review identified seven ALK-positive ALCL with foci in the posterior fossa. Interestingly, although ALK-inhibition therapy with second- and third-generation drugs has shown some potential in ALK-positive, non-CNS lymphomas [78], none of the identified patients received ALK-inhibition therapy.

### 4.4. Intracranial Mesenchymal Tumors That Are FET::CREB-Fusion-Positive

IMMTs, now categorized under the “Intracranial mesenchymal tumor, FET::CREB-fusion-positive” category of the 2021 WHO Classification of Tumors of the Central Nervous System [50], are tumors of mesenchymal, nonmeningothelial origin with varying morhpological characteristics [79]. They are mainly found in extra-axial and supratentorial locations, often within the ventricular system or attached to the dura or meninges. Posterior fossa localization is less common, for example, in the cerebellopontine angle [80]. In our review, we identified only two cases of ALK-positive, posterior fossa tumors characterized as IMMTs [58,59]. The authors of the aforementioned case reports did not discuss potential implications of ALK-positivity in this type of tumor. 

### 4.5. Inflammatory Myofibroblastic Tumors

IMTs are rare lesions of proliferating myofibroblastic cells mixed with inflammatory cells, such as lymphocytes, plasma cells, and eosinophils. These tumors can occur in various parts of the body but are most commonly found in the lungs, abdominal, and pelvic regions. Intracranial localization is extremely rare, and the diagnosis of such cases has been hindered due to overlapping clinical and pathological features shared between non-neoplastic inflammatory processes and IMTs. While approximately 70% of all IMT cases show ALK fusions [81], predominantly RANBP2–ALK [82], it is notable that most CNS IMT cases documented in the literature lack ALK expression [83]. However, it should be noted that, in the biggest case series of CNS lesions, the authors included both neoplastic and non-neoplastic lesions [83]. Nevertheless, case reports of ALK-positive IMT with CNS localization do exist [84,85]. In our review, we identified only one IMT with posterior fossa localization that was ALK negative with immunohistochemistry but exhibited ALK translocation when examined with FISH [62].

Given that ALK inhibitors have shown efficacy in extracranial IMT [86,87], the recent Pediatric Strategy Forum for ALK inhibition in pediatric malignancies suggested that performing molecular analysis in IMT could aid in the identification of specific fusions [86]. By expanding this logic, this strategy should also be followed in intracranial IMT and the choice of ALK inhibitor should favor drugs with higher CNS penetration. On that note, Chennouf et al. described a patient with ALK rearranged IMT of the CNS who was successfully treated with crizotinib, a first-line ALK inhibitor. This treatment was particularly effective post-radiotherapy, possibly due to enhanced blood–brain barrier permeability and led to a marked partial response in the patient. This suggests that for ALK rearranged intracranial IMT, a combined approach of ALK inhibition and radiotherapy could be a highly effective treatment regimen, capitalizing on the synergistic effect of these therapies to overcome the limited drug penetration across the blood–brain barrier [88]. 

### 4.6. Therapeutic Considerations

The available evidence regarding the blood–brain barrier penetration of ALK inhibitors mainly stems from studies investigating their use in NSCLC brain metastases. The first-generation ALK inhibitor Crizotinib has demonstrated some intracranial efficacy in treating NSCLC CNS metastases, as seen in the PROFILE 1014 study, but its overall effectiveness in the CNS is considered limited [89,90]. In contrast, second-generation ALK inhibitors, including alectinib, brigatinib, and ceritinib, have shown improved outcomes. Alectinib demonstrated superior efficacy in the Phase III ALEX trial, significantly improving progression-free survival and response rates compared to crizotinib in ALK-positive NSCLC patients [91,92]. In the Japanese J-ALEX trial, alectinib showed a lower risk of CNS progression, highlighting its effectiveness in patients with brain metastases [93]. Additionally, studies like ALUR and a pooled analysis of two phase-II trials underscored alectinib’s higher intracranial disease control rates and prolonged median duration of CNS response [94,95,96]. Moving to the third generation, Lorlatinib was specifically designed for better CNS activity and showed substantial efficacy in mouse models of brain metastases [97,98]. It stands out with its high intracranial activity, achieving an intracranial objective response rate of up to 87% in specific populations enrolled in early clinical trials [99,100]. 

Given the demonstrated efficacy of ALK inhibitors, particularly second- and third-generation agents, in penetrating the blood–brain barrier and controlling CNS metastases in NSCLC, it could be hypothesized that these drugs can treat primary intracranial tumors with ALK alterations/expression, including those in the posterior cranial fossa. This potential is particularly intriguing for Lorlatinib, whose design specifically targets CNS activity and might offer substantial therapeutic benefits in primary brain tumors with ALK involvement. 

A recent article by Rigaud et al. documents the use of ceritinib, lorlatinib, and alectinib in a pediatric population with CNS relapse or progression of ALK-positive ALCL [17]. Notably, a rapid and profound response was observed in almost all patients, with the majority achieving complete remission. This study thus highlights promising clinical and radiological responses due to these next-generation inhibitors in CNS cases [17]. This finding aligns with the hypothesis of the therapeutic potential of ALK inhibitors in primary intracranial tumors, mentioned earlier.

Interestingly, there are ongoing clinical trials evaluating the use of ALK inhibitors in various solid tumors and hematological malignancies (NCT05384626, NCT04925609). If these studies enroll patients with intracranial malignancies, especially posterior fossa tumors, then they could shed light on the efficacy of ALK inhibitors in these tumors.

### 4.7. Limitations

The main limitation of the current scoping review derives from the scarce evidence available in the literature regarding ALK in tumors of the posterior fossa, mostly in the form of case reports. Nevertheless, the available evidence was reviewed and presented in a comprehensive manner and novel perspectives have been identified and presented. 

## 5. Conclusions

The present comprehensive scoping review has provided an overview on the presence of ALK expression and of ALK genetic alterations in various non-glial tumors of the posterior cranial fossa, a site associated with increased surgical morbidity and mortality. 

Analysis of ALK expression holds significant value in medulloblastoma, particularly for identifying WNT-activated tumors. It serves as a diagnostic tool, differentiating WNT medulloblastomas from other pediatric brain tumors. Furthermore, ALK is associated with a favorable prognosis in medulloblastoma, contrary to its adverse prognostic implication in other pediatric cancers like neuroblastoma. The consistent reporting of ALK expression in CNS histiocytosis, especially within the posterior fossa, points to a topographical predilection. Moreover, a therapeutic potential of ALK inhibitors in histiocytosis is supported by a limited number of observations that warrant further investigation. Additional ALK-positive/altered tumors that have been previously localized within the posterior fossa, include anaplastic large-cell lymphomas, IMT, and intracranial mesenchymal tumors that are FET::CREB-fusion-positive.

## Data Availability

The data presented in this study are available in the text and tables of the manuscript.

## Supplementary Materials

The following supporting information can be downloaded at: https://www.mdpi.com/article/10.3390/cancers16030650/s1, Supplementary File S1: Exact algorithms used for database search; Supplementary File S2: PRISMA checklist for scoping reviews of the literature.
